# Anti-inflammatory activities of *Sigesbeckia glabrescens* Makino: combined in vitro and in silico investigations

**DOI:** 10.1186/s13020-019-0260-y

**Published:** 2019-09-23

**Authors:** Zhangfeng Zhong, Qianru Zhang, Hongxun Tao, Wei Sang, Liao Cui, Wenan Qiang, Wai San Cheang, Yuanjia Hu, Hua Yu, Yitao Wang

**Affiliations:** 1 Institute of Chinese Medical Sciences, State Key Laboratory of Quality Research in Chinese Medicine, University of Macau, Macao, China; 2 HKBU Shenzhen Research Center, Shenzhen, Guangdong China; 3 School of Chinese Medicine, Hong Kong Baptist University, Kowloon Tong, Hong Kong China; 4 Guangdong Key Laboratory for Research and Development of Natural Drugs, Guangdong Medical University, Zhanjiang, China; 5 School of Pharmacy, Zunyi Medical University, Zunyi, Guizhou China; 6 Center for Developmental Therapeutics, Chemistry of Life Processes Institute, Northwestern University, Evanston, IL USA; 7 Devision of Reproductive Science in Medicine, Department of Obstetrics and Gynecology, Feinberg School of Medicine, Northwestern University, Chicago, IL USA

**Keywords:** *Sigesbeckia glabrescens* Makino, Inflammation, Lipopolysaccharide, Docking, TLR4, NF-κB

## Abstract

**Background:**

*Sigesbeckia glabrescens* Makino (SG) is one of the important plant origins of Sigesbeckiae herba and has been widely used for the treatment of chronic inflammatory diseases in China. However, the underlying anti-inflammatory mechanism of SG is rarely investigated and reported. There are more than 40 kinds of chemical constituents in SG, but the action of the bioactive compounds of SG is still unclear. Therefore, we aimed to systemically investigate the mechanisms behind the anti-inflammatory properties of SG by combining in vitro and in silico investigations.

**Methods:**

Cytotoxicity was measured using the 3-[4,5-dimethyl-2-thiazolyl]-2,5-diphenyltetrazolium bromide (MTT) and lactate dehydrogenase (LDH) assays. Nitric oxide (NO) release was detected using the Griess assay. The secretion of pro-inflammatory cytokines and the expression of relevant proteins were assessed using ELISA kits and Western blots, respectively. Molecular docking was performed and scored using AutoDock via a comparison with the molecular docking of *N*-acetyl-d-glucosamine (NAG).

**Results:**

In lipopolysaccharides (LPS)-stimulated macrophages, SG significantly inhibited NO, MCP-1, and IL-6 secretion; iNOS expression; and NF-κB activation but did not significantly affect MAPK signalling (p38, ERK, and JNK). Moreover, the results from the molecular docking prediction suggested that over 10 compounds in SG could likely target TLR4, p105, and p65.

**Conclusions:**

These findings suggest that the anti-inflammatory effects of SG are highly related to the inactivation of NF-κB. Moreover, this study provides a novel approach to investigate the effects of herbal medicine using combined in vitro and in silico investigations.

## Background

Inflammation is a complex physiological response against harmful stimuli, including microorganisms, dust particles, damaged self‑cells or external irritants [1]. Inflammatory responses are characterized by an influx of white blood cells leading to redness, pain, and swelling; this response is associated with various chronic diseases, such as diabetes, obesity, inflammatory bowel disease, asthma, arthritis, cardiovascular disease, and cancer [2]. Cyclooxygenase-2 (COX-2) and inducible nitric oxide synthase (iNOS) have been recognized as inflammatory markers in many studies, which have observed that the expression of these markers could be induced by various pro-inflammatory factors, including lipopolysaccharides (LPS) and tumour necrosis factor-α (TNF-α) [3]. iNOS was also found to possibly enhance COX-2 catalytic activity, resulting in an abundance of its enzymatic product, prostaglandin E 2 (PGE2). Furthermore, nitric oxide (NO) could be generated by iNOS, which has also been found in multiple inflammatory conditions [4]. The mitogen-activated protein kinase (MAPK) signalling pathway is involved in this crosstalk between iNOS and COX‑2, which has a critical role in the control of cell responses to cytokines and stresses [5]. The pathway could induce both the transcriptional and post-transcriptional activations of COX-2.

Nuclear factor kappa-light-chain-enhancer of activated B cells (NF-κB), a transcription factor, promotes immunity by controlling genes and is involved in the inflammatory response of most cell types. Cytokines can stimulate cell surface receptors, such as toll-like receptors (TLRs), to activate the NF-κB signalling cascade [6]. In general, NF-κB can be activated by the phosphorylation of inhibitor κB (IκB), which results in IκB degradation; this is followed by NF-κB translocation to the nucleus where it upregulates the expression of pro-inflammatory genes [7]. NF-κB inactivation was associated with the inhibition of IκB degradation and the decrease of the nuclear p65 and p50 levels.

*Sigesbeckia glabrescens* Makino (SG) belongs to the genus Sigesbeckia and is widely distributed in China, Japan, and Korea. SG has been traditionally used for the management of chronic diseases, including arthritis, the numbness of limbs and hemiplegia, and it is known to originate from the plant, Sigesbeckiae herba. Based on the current phytochemical studies, the main chemicals in SG include flavonoids, sesquiterpenoids, diterpenoids, triterpenoids, sterols, and additional components [8]. Among them, kaurane-type and pimarane-type diterpenoids, including siegeskaurolic acid, kirenol, and sesquiterpene lactone, are the main active ingredients in SG. Pharmacologically, the SG-derived extracts and/or purified components have been reported to possess various pharmacological activities, including anti-inflammatory [9], anti-bacterial [10], and anti-cancer effects [11].

Although anti-inflammation is one of the most important therapeutic effects of SG, the potential biological mechanisms of SG on immunity are still not recognized and need to be further investigated. To reduce the cost and increase the efficiency of drug discovery, the application of in silico approaches can enrich effective compounds among SG and adequately indicate the underlying mechanism of anti-inflammatory action of SG [12].

## Methods

### Chemical and reagents

Kirenol and darutoside were purchased from Chengdu Pufei De Biotech Co., Ltd. (Chengdu, China). Acetonitrile (ACN, HPLC grade) and methanol (HPLC grade) were purchased from RCI Labscan Limited (Pathumwan, Bangkok, Thailand). Phosphoric acid (analytical grade) was purchased from Sigma Chemicals Ltd. (St. Louis, MO, USA). Milli-Q water was prepared using a Milli-Q system (Millipore, MA, United States).

Dulbecco’s modified Eagle’s medium (DMEM) was purchased from Gibco (Gaithersburg, MD, United States). Foetal bovine serum (FBS), phosphate-buffered saline (PBS), penicillin-streptomycin (PS), 0.25% (w/v) trypsin/1 mM EDTA, and Lipofectamine 2000 were obtained from Invitrogen (Carlsbad, CA, United States). 3-[4,5-dimethyl-2-thiazolyl]-2,5-diphenyltetrazolium bromide (MTT) and LPS (*Escherichia coli* O111:B4, Purified by Phenol extraction, PREMIUM) were supplied by Sigma-Aldrich (St. Louis, MO, United States). IL-6, MCP-1, and TNF-α immunoassay kits were purchased from NeoBioscience (NeoBioscience, Shenzhen, China). Radioimmunoprecipitation assay (RIPA) lysis buffer was obtained from Santa Cruz (Santa Cruz, CA, United States). Primary antibodies against COX-2, p-p38 (Thr180/Tyr182), p38, p-ERK1/2 (Thr202/Tyr204), ERK1/2, p-JNK (Thr183/Tyr185), JNK, KEAP1, p-PIK3C3 (Ser249), PIC3C3, iNOS, p-p65 (Ser536), p65, p-IKK α/β (Ser176/180), IKK-β, p-IκBα (Ser32), IκBα, GAPDH, as well as the secondary antibodies, were purchased from Cell Signaling Technology (Danvers, MA, United States). The pNF-κB-luc reporter gene was purchased from Beyotime (Beyotime, Shanghai, China). The pRL-TK vector and dual-luciferase reporter assay system were obtained from Promega Corporation (Madison, WI, USA).

### Preparation of SG extract

The herbal material of SG was collected from Jinyun (Zhejiang province, China) and authenticated by the corresponding author. The voucher specimens (No. SG-017) were deposited at the Institute of Chinese Medical Sciences, University of Macau, Macao, China.

Herbs from SG (100 g) were reflux-extracted twice with 50% ethanol (1:10, w/v) for 1 h each. The combined extracts were filtered with filter paper after cooling and were then concentrated under reduced pressure to remove the ethanol. The powdered SG extract (yield: 22.2%) was obtained by lyophilizing the concentrated samples with a VirTis Freeze Dryer (The VirTis Company, New York, USA).

### Liquid chromatography

The quantification of kirenol and darutoside in SG extract was performed using a Waters ACQUITY UPLC System (Waters Corp., Milford, MA, USA). Separation was achieved with an ACQUITY UPLC HSS T3 column (2.1 mm × 150 mm I.D., 1.8 μm) at 40 °C. Elution was performed with the mobile phase of A (0.2% phosphoric acid in water) and B (0.2% phosphoric acid in ACN) under a buffer gradient of 17% B for the first 5 min, an increase to 19% B for 5 min, 35% B for 10 min, 50% B for 10 min and then 100% B for 10 min. The flow rate was 0.4 mL/min, and the injection volume was 2 μL. The analytes were monitored at a UV wavelength of 215 nm. Prior to the next injection, the column was washed with 100% B for 5 min and was equilibrated with the initial mobile phase (17% B) for 5 min.

### Cell culture and drug treatment

The RAW 264.7 cell line (mouse leukemic monocyte macrophages) was obtained from the American Type Culture Collection (ATCC, Manassas, VA, USA) and was cultured in DMEM containing 10% FBS, penicillin (100 U/mL) and streptomycin (100 mg/mL). The cells were incubated in a 5% CO_2_ humidified incubator at 37 °C and were passaged twice a week.

### Cell density assay

RAW 264.7 cells were treated with SG (0, 50, 100, 200, 400, and 800 μg/mL) for 24 h. To observe the cell density, the cell images were captured using a microscope (Olympus MVX10, Japan) equipped with a digital camera (ColorView II, Soft Imaging System, Olympus) under 100× magnification. Representative images were taken from at least three independent experiments.

### Cell viability assay

The cell viability of RAW 264.7 cells was determined by an MTT assay as described previously [13]. In brief, the exponentially growing cells were plated in 96-well plates at a density of 1 × 10^4^/well. After the cells were treated with SG (0, 50, 100, 200, 400, and 800 μg/mL) for 24 h, the MTT reaction solution (20 µL, 5 mg/mL) was added to each well. The absorbance values of the formazans dissolved in DMSO at 570 nm were determined using the SpectraMax M5 microplate reader (Molecular Devices, Silicon Valley, CA, United States).

### Cell cytotoxicity assay

LDH release from RAW 264.7 cells was determined with a cytotoxicity detection kit (Roche) according to a previous publication [14]. In brief, the 96-well culture plate was centrifuged at 350×*g* for 5 min after the cells were induced with the desired drug treatment. The supernatants (50 µL) were taken from each well and reacted with the LDH substrate. The absorbance values at 490 and 600 nm were recorded using the SpectraMax M5 microplate reader (Molecular Devices, Silicon Valley, CA, United States).

### CFDA-SE cell proliferation assay

Regarding the CFDA-SE fluorescent characterization, cell proliferation was determined with the CFDA-SE probe (Molecular Probes) using flow cytometry (BD FACS Canto™, BD Biosciences, San Jose, CA, United States). In brief, RAW 264.7 cells were plated in 6-well plates at a density of 5 × 10^2^/well and were loaded with CFDA-SE probe according to the manufacturer’s protocol. The cells were harvested and washed with PBS after treatment with SG (0, 25, 50, 100, 200, and 400 μg/mL) for 6 days. The fluorescence shift was represented using FlowJo software (TreeStar, Ashland, OR, United States).

### NO release assays

RAW 264.7 cells were seeded into 96-well plates at a density of 1 × 10^4^/well. Based on the characterization of herbal medicine and our preliminary studies on this model, the cells were pretreated with SG (0, 25, 50, 100, and 200 μg/mL) for 4 h followed by the addition of LPS (1 μg/mL) for 12 h. The NO release from the culture media was collected and detected using the Griess reagent according to the manufacturer’s protocol. Based on the background of NO release from the uninduced cells, the inhibitory effect of SG on NO release was calculated using the following formula: Inhibitory rate (%) = (LPS group − treatment group)/(LPS group − negative group) * 100%.

### ELISA assay

RAW 264.7 cells were seeded into 24-well plates at a density of 5 × 10^4^/well. The cells were pretreated with SG (0, 50, 100, and 200 μg/mL) for 4 h followed by the addition of LPS (1 μg/mL) for 12 h. The conditioned culture media was collected, and the release of IL-6, MCP-1, and TNF-α from the cells was detected with an immunoassay kit (NeoBioscience, Shenzhen, China) according to the manufacturer’s protocol. The absorbance values at 450 and 570 nm were determined using the SpectraMax M5 microplate reader (Molecular Devices, Silicon Valley, CA, United States).

### Western blotting assay

Western blotting assays were conducted according to previous studies [15]. In brief, RAW 264.7 cells were pretreated with SG (0, 25, 50, and 100 μg/mL) for 4 h followed by the addition of LPS (1 μg/mL) for 12 h. The cells were harvested, and the total proteins were extracted with RIPA lysis buffer after the desired drug treatment. The proteins were transferred onto a PVDF membrane after being separated by SDS-PAGE. The membrane was blocked with non-fat milk followed by the incubation of the appropriate primary antibodies and the corresponding second antibodies. The specific protein bands were visualized with an Amersham ECL™ Advance Western Blotting Detection Kit (GE Healthcare Life Sciences, Uppsala, Sweden).

### Dual-luciferase reporter assay

RAW 264.7 cells were plated into a 24-well plate at a density of 5 × 10^4^/well. The cells were co-transfected with 0.8 µg pNF-kB-luc and 0.8 µg pRL-TK as a transfection efficiency control. The plasmids and the TurboFect transfection reagents were mixed in Opti-MEM serum-free medium for a 20-min incubation at 25 °C. The mixture of DNA-TurboFect (100 µL) was transferred into each well. After an overnight incubation, the cells were cultivated with fresh completed medium for 48 h. The cell lysates were collected and were reacted with the substrate according to the dual-luciferase assay protocol [16]. The sample light output was observed using the SpectraMax M5 microplate reader (Molecular Devices, Silicon Valley, CA, United States). The data were aligned to the pRL-TK values prior to normalization with the appropriate control.

### Molecular docking assay

SG compounds were retrieved from four chemical databases, including SciFinder, Combined Chemical Dictionary v2009 (CCD), Dictionary of Natural Products (DNP, http://dnp.chemnetbase.com/), and the Chinese Academy of Sciences’ Chemistry Database (CASCD, http://www.organchem.csdb.cn/, [1978–2016]). The molecular docking simulation for SG compounds and TLR4 (PDB ID: 3FXI), p65 (PDB ID: 1NFI), and p105 (PDB ID: 1SVC) were performed and scored using AutoDock Vina by comparison with that of *N*-acetyl-d-glucosamine (NAG) [17]. The crystal structures of TLR4 (PDB ID: 3FXI), p65 (PDB ID: 1NFI), and p105 (PDB ID: 1SVC) were derived from the RCSB Protein Data Bank [18–20]. When in the dock the screen, NAG was utilized as an inhibitor for defining the threshold of binding affinity that SG compounds docking with three targeting proteins. The thresholds that NAG docking with TLR4, p105, and p65, were − 6.9, − 5.5, and − 6.0 kcal/mol, respectively.

### Statistical analysis

All data represent the mean of three separately performed experiments plus or minus the standard deviation or the standard error of the mean. The significance of the differences between the groups was evaluated by one-way ANOVA using GraphPad Prism software (GraphPad Software, United States). Newman–Keuls multiple comparison tests were performed for post hoc pairwise comparisons. *P*-values less than 0.05 were considered significant.

## Results

### Quantitative analysis of kirenol and darutoside in SG extract

**Fig. 1:**
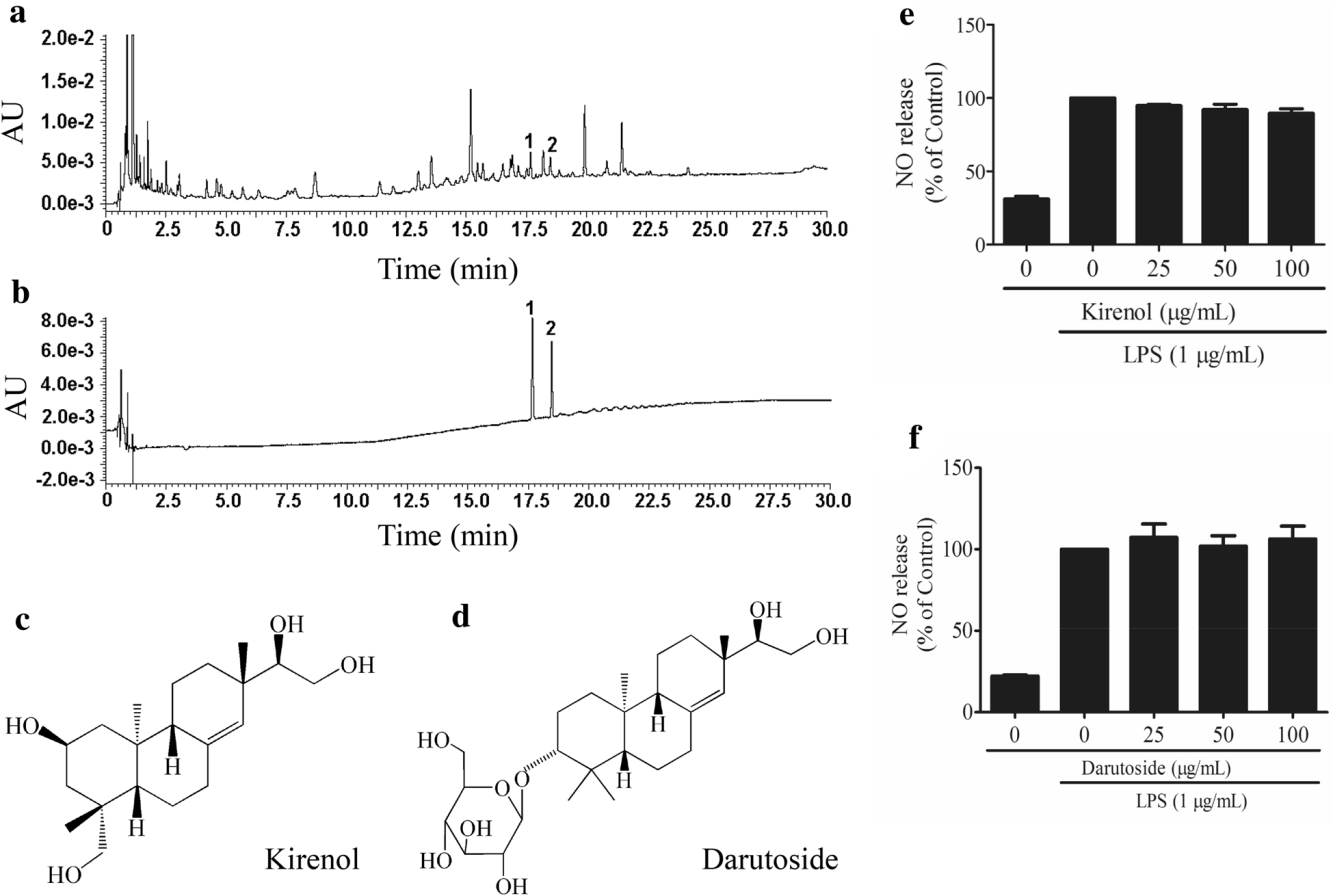
Quantitative analysis of kirenol and darutoside in *Sigesbeckia glabrescens* Makino (SG). HPLC chromatograms of **a** SG extract (1 mg/mL) and **b** mixed standards (7.5 μg/mL of kirenol and darutoside). **1**: kirenol; **2**: darutoside. **c** Chemical structure of kirenol. **d** Chemical structure of darutoside. RAW 264.7 cells were pretreated with **e** kirenol (0, 25, 50, and 100 μg/mL) or **f** darutoside (0, 25, 50, and 100 μg/mL) for 4 h followed by the addition of lipopolysaccharides (LPS, 1 μg/mL) for 12 h. Nitric oxide (NO) release was detected using the Griess reagent

The chromatograms of SG extract (1 mg/mL) and mixed standards (7.5 μg/mL of kirenol and darutoside) are illustrated in Fig. 1a, b. The contents (%, w/w) of kirenol (Fig. 1c) and darutoside (Fig. 1d) in SG extract were determined to be 0.33 ± 0.02% and 0.47 ± 0.03%, respectively. Moreover, kirenol (Fig. 1e) and darutoside (Fig. 1f) were unable to attenuate the NO release induced by LPS in RAW 264.7 cells.

### Effects of SG on cell number, viability, cytotoxicity, and proliferation in RAW 264.7 cells

**Fig. 2:**
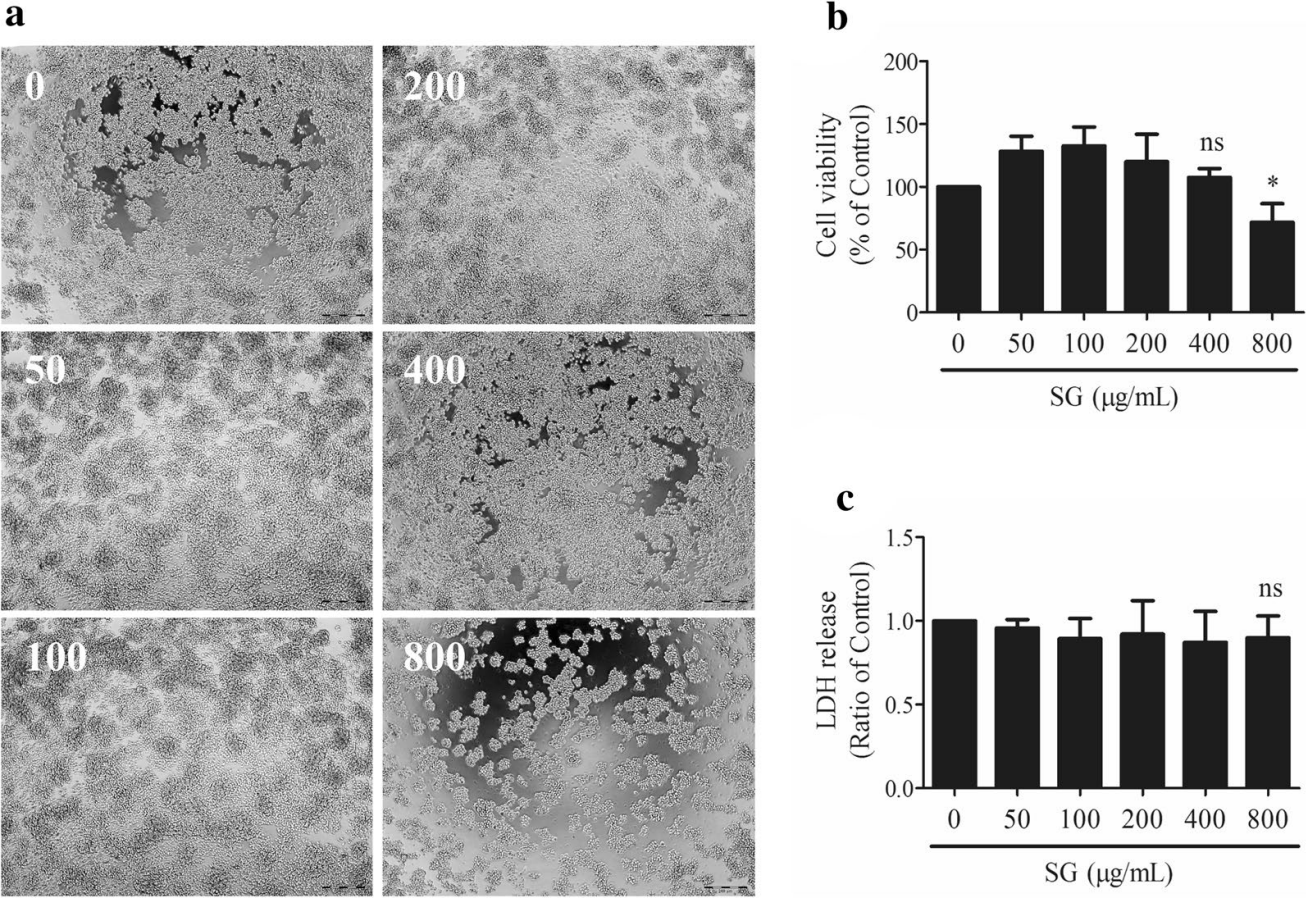
Effects of *Sigesbeckia glabrescens* Makino (SG) on **a** cell density, **b** cell viability, and **c** cell cytotoxicity. RAW 264.7 cells were treated with SG (0, 50, 100, 200, 400, and 800 μg/mL) for 24 h. *P < 0.05 vs. control, ns stands for not statistically significant

**Fig. 3:**
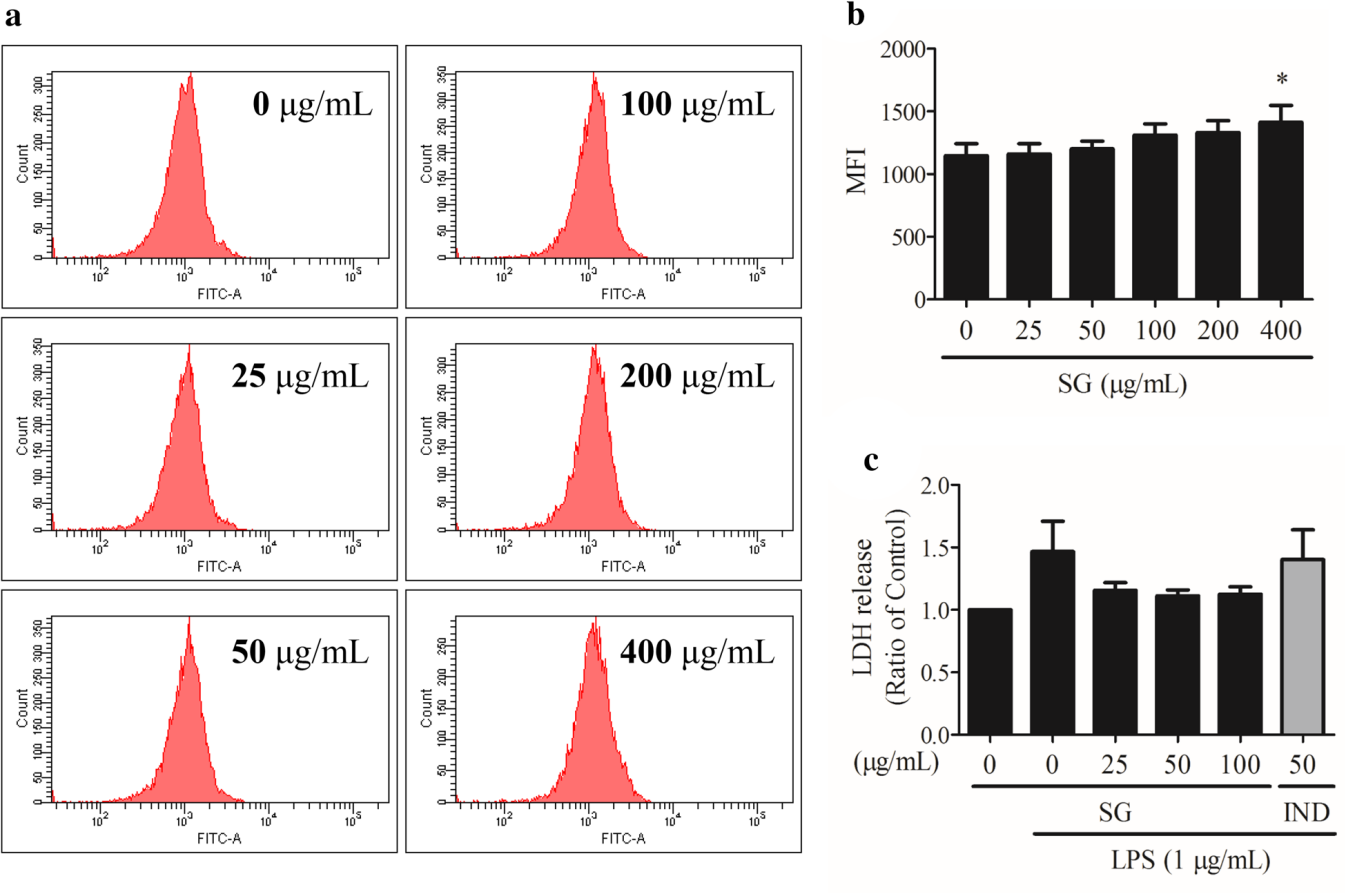
Effects of *Sigesbeckia glabrescens* Makino (SG) on cell proliferation and cell cytotoxicity. **a** RAW 264.7 cells were treated with SG (0, 25, 50, 100, 200, and 400 μg/mL) for 6 days. Cell proliferation was determined with the CFDA-SE probe using flow cytometry. **b** Statistical results of **a**. **c** RAW 264.7 cells were pretreated with SG (0, 25, 50, and 100 μg/mL) for 4 h followed by the addition of lipopolysaccharides (LPS, 1 μg/mL) for 12 h. Indomethacin (IND) was selected as a positive drug. *P < 0.05 vs. control

To rule out the false positive anti-inflammatory effect of SG, cell density assays, cell viability assays, cell cytotoxicity assays, and CFDA-SE assays were performed to select the non-toxic concentrations of SG. In the photo capture and MTT assays, the SG extract decreased the cell number (Fig. 2a) and inhibited the cell viability (Fig. 2b) of RAW 264.7 cells at a concentration of 800 µg/mL compared to the vehicle control group. LDH release from the RAW 264.7 cells remained unchanged after 24 h of treatment with SG extract, even at the high concentration of 800 µg/mL (Fig. 2c). In the CFDA-SE staining assay, the mean fluorescence intensity induced by SG extract (400 µg/mL) was significantly stronger than the vehicle control after treatment for 6 days (Fig. 3a, b). As shown in Fig. 3c, LPS (1 µg/mL) was unable to enhance the LDH release from RAW 264.7 cells when induced by SG extract (25, 50, 100, or 200 µg/mL). Therefore, in consideration of the effects of SG extract on the cell number, viability, cytotoxicity, and proliferation, a series of non-toxic concentrations (25, 50, 100, or 200 µg/mL) was selected for further studies investigating the anti-inflammatory effects of SG.

### SG inhibited the LPS-induced NO and cytokine release in RAW 264.7 cells

**Fig. 4:**
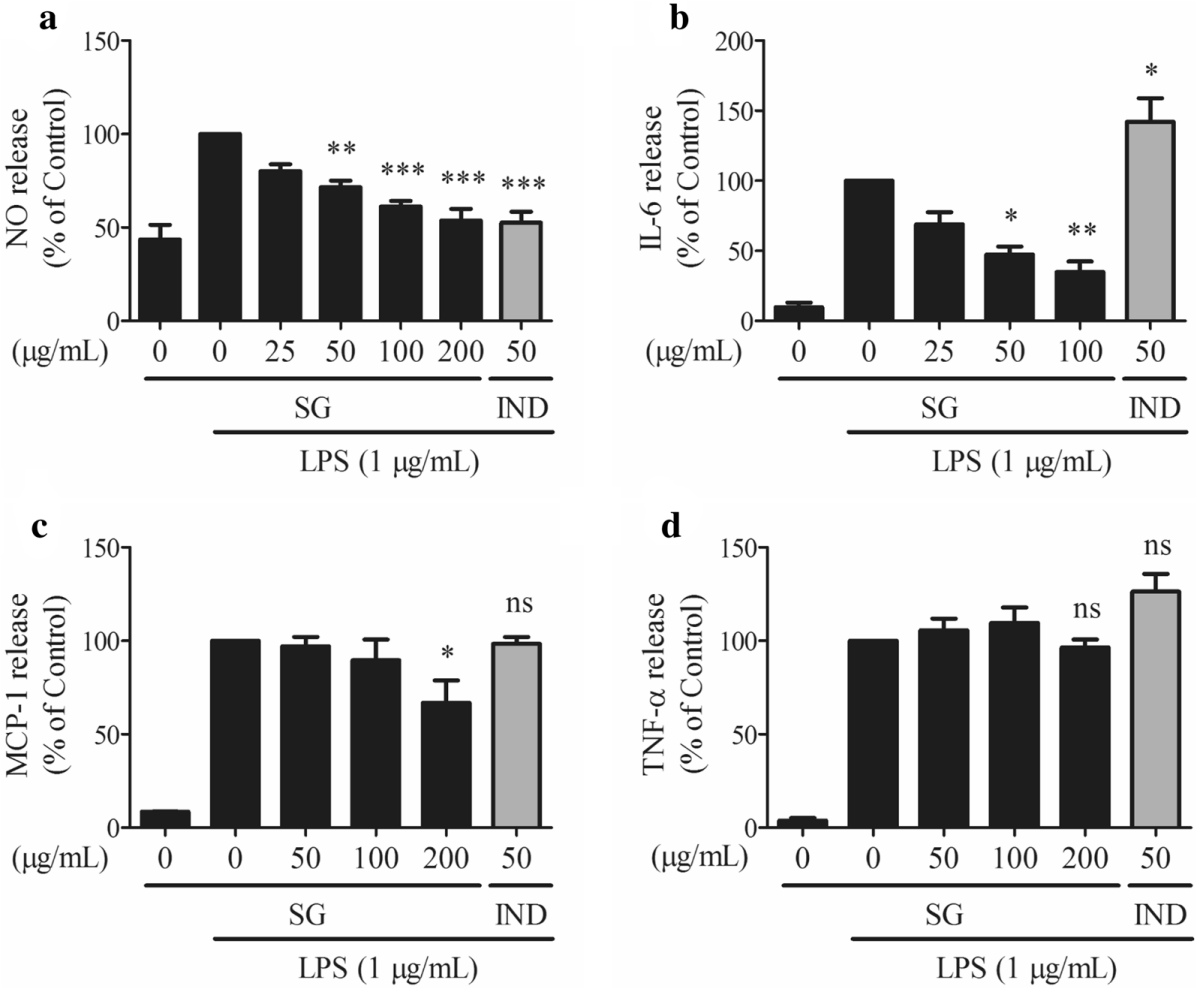
Effects of *Sigesbeckia glabrescens* Makino (SG) on the secretions of **a** nitric oxide (NO), **b** IL-6, **c** MCP-1, and **d** TNF-α. RAW 264.7 cells were pretreated with SG (0, 25, 50, 100 or 200 μg/mL) for 4 h followed by the addition of lipopolysaccharides (LPS, 1 μg/mL) for 12 h. Indomethacin (IND) was selected as a positive drug. *P < 0.05 vs. LPS-induced cells, **P < 0.01 vs. LPS-induced cells, ***P < 0.001 vs. LPS-induced cells, and ns stands for not statistically significant

To investigate the anti-inflammatory effects of SG, Griess reaction assays and ELISAs were performed. As shown in Fig. 4a, SG extract inhibited the NO release induced by LPS in RAW 264.7 cells in a dose-dependent manner. LPS-induced NO release was suppressed by 82.09% after SG extract treatment at a concentration of 200 µg/mL, as well as indomethacin (IND). In the ELISA, SG extract was the most effective to inhibit the IL-6 secretion induced by LPS (Fig. 4b) and was moderately effective in inhibiting MCP-1 secretion induced by LPS (Fig. 4c) in RAW 264.7 cells. However, LPS-induced TNF-α secretion could not be suppressed by SG extract under the same conditions (Fig. 4d). Moreover, indomethacin could not effectively inhibit IL-6, MCP-1, and TNF-α release. The results indicate that TNF-α is likely non-specific target of SG.

### The underlying mechanism of the anti-inflammatory effect of SG in RAW 264.7 cells

**Fig. 5:**
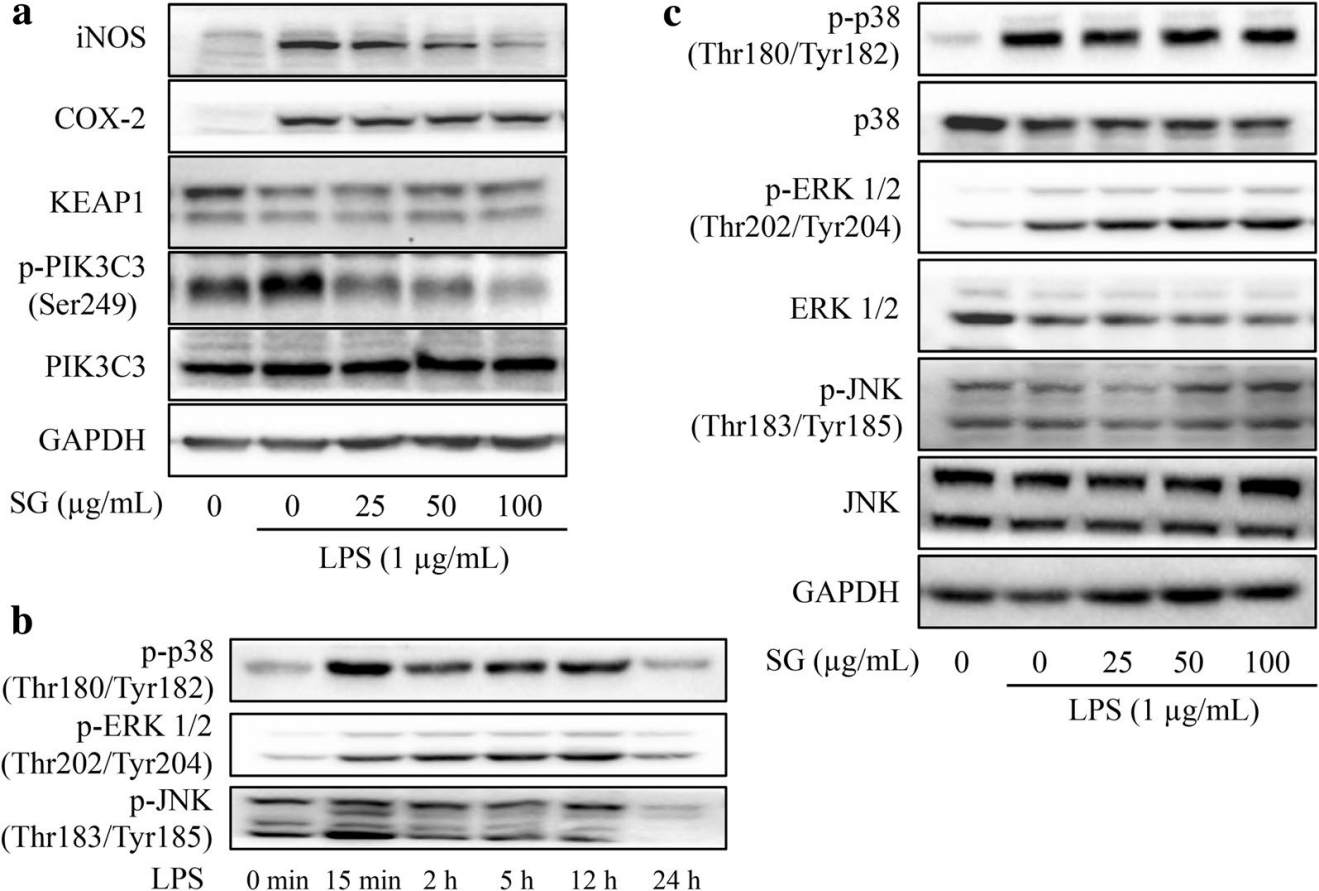
Effects of *Sigesbeckia glabrescens* Makino (SG) on the relevant pathways. RAW 264.7 cells were pretreated with SG (0, 25, 50, and 100 μg/mL) for 4 h followed by the addition of lipopolysaccharides (LPS, 1 μg/mL) for 12 h. iNOS pathway (**a**) and the timing assay (**b**) for the MAPK pathway (**c**) were evaluated by Western blotting

**Fig. 6:**
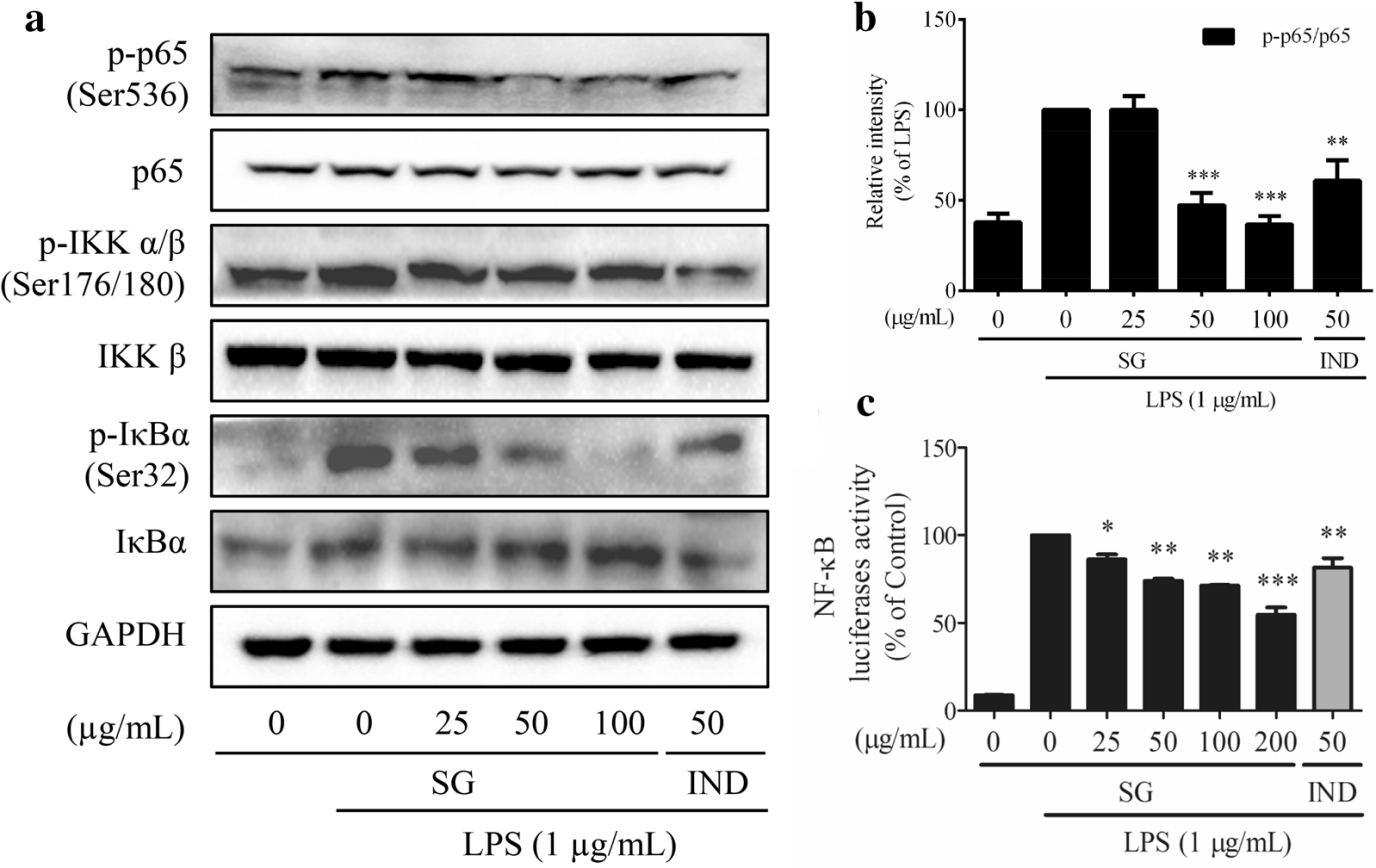
Effects of *Sigesbeckia glabrescens* Makino (SG) on the relevant pathways. RAW 264.7 cells were pretreated with SG (0, 25, 50, 100 or 200 μg/mL) for 4 h followed by the addition of lipopolysaccharides (LPS, 1 μg/mL) for 12 h. The NF-κB pathway was investigated by **a**, **b** Western blotting (p-p65, Ser536) and **c** a dual-luciferase reporter assay. Indomethacin (IND) was selected as a positive drug. *P < 0.05 vs. LPS-induced cells, **P < 0.01 vs. LPS-induced cells, and ***P < 0.001 vs. LPS-induced cells

To investigate the underlying mechanism of the anti-inflammatory effects of SG, a Western blotting assay was performed. SG extract was more potent in suppressing the LPS-induced iNOS expression than the COX-2 expression. SG extract potentially restored the LPS-induced decrease in KEAP1 expression (Fig. 5a). We found that MAPK phosphorylation/activation occurred after stimulation of a few minutes to 12 h. To observe the real balancing effect of SG on the MAPK pathway, the timing was selected at 12 h after rigorous preliminary studies on the model of LPS-induced RAW 264.7 cells (Fig. 5b). Otherwise, SG extract inhibited the phosphorylation of PIK3C3 at Ser249, IκBα at Ser32, and p65 at Ser536 induced by LPS in RAW 264.7 cells, but SG extract had no effects on the phosphorylation of ERK1/2, p38, JNK, and IKKα/β (Figs. 5a, c, 6a, b). Furthermore, a dual-luciferase reporter assay was performed to confirm the regulatory effects of SG on the NF-κB pathway. LPS significantly increased the NF-κB transcriptional activity in RAW 264.7 cells compared to that in unstimulated cells. However, SG extract attenuated the NF-κB-driven luciferase activity in LPS-stimulated cells, with SG being slightly more potent than indomethacin in this assay (Fig. 6c). Therefore, SG exhibits anti-inflammatory effects via a MAPK-independent pathway in RAW 264.7 cells. However, NF-κB may play an important role in the regulation of SG that functions in inflammation.

### Docking of the compounds from SG to TLR4, p105, and p65

SG contains complex constituents, which is not easy to understand with limited analyses and biological technologies. A molecular docking assay was performed to predict the compound-targeting interaction. To screen the docked phytochemicals, a threshold of − 6.9 kcal/mol was applied to the compound-TLR4 interaction, a threshold of − 5.5 kcal/mol was applied to the compound-p105 interaction, and a threshold of − 6.0 kcal/mol was applied to the compound-p65 interaction. The results of molecular docking showed that 10, 12, and 11 kinds of compounds from SG could bind with TLR4 (Table 1), p105 (Table 2), and p65 (Table 3), respectively, at hydrophobic pockets, which were the binding sites of *N*-acetyl-d-glucosamine (NAG). Daucosterol, darutoside, and 8 other kinds of compounds were predicted to likely target TLR4, p105, and p65. Therefore, the docking results suggest that SG exhibits anti-inflammatory effects via targeting the TLR4 and NF-κB pathways.

**Table 1:** Docking results of compounds from *Sigesbeckia glabrescens* Makino (SG) with toll-like receptor 4 (TLR4)

Compound name	Pubchem_ID	Affinity (kcal/mol)	References
Darutoside	44715524	− 8.9	[34]
3,7-Di-*O*-methylquercetin	5280417	− 8.2	[23]
Daucosterol	5742590	− 8.1	[8]
3-*O*-Methylquercetin	5280681	− 8	[23]
β-Sitosterol	222284	− 7.8	[35]
5-Hydroxy-2-(3-hydroxy-4-methoxyphenyl)-3,7-dimethoxy-4H-chromen-4-one	–	− 7.7	[23]
Quercetin 3,4′-dimethyl ether	5380905	− 7.6	[23]
NPDAQZUAWDTCIZ-UHFFFAOYSA-N	53462154	− 7.5	[34]
Darutigenol	3037565	− 7.4	[34]
8(14)-Pimarene-2,15,16,19-tetrol;(ent-2,15S)-form,16-Ac	–	− 7	[36]
Kirenol (f)	15736732	− 6.8	[8]
Ferulic acid (f)	445858	− 5.7	[35]
Succinic acid (f)	1110	− 4.5	[35]
Heptacosanol (f)	74822	− 4	[35]

f indicates that the compound failed to interact with the known receptor based on docking simulation

**Table 2:** Docking results of compounds from *Sigesbeckia glabrescens* Makino (SG) with p105

Compound name	Pubchem ID	Affinity (kcal/mol)	References
Daucosterol	5742590	− 7.4	[8]
3,7-Di-*O*-methylquercetin	5280417	− 7.1	[23]
Darutoside	44715524	− 6.8	[34]
NPDAQZUAWDTCIZ-UHFFFAOYSA-N	53462154	− 6.8	[34]
Darutigenol	3037565	− 6.7	[34]
β-Sitosterol	222284	− 6.6	[35]
8(14)-Pimarene-2,15,16,19-tetrol;(ent-2,15S)-form,16-Ac	–	− 6.5	[36]
3-*O*-Methylquercetin	5280681	− 6.5	[23]
5-Hydroxy-2-(3-hydroxy-4-methoxyphenyl)-3,7-dimethoxy-4H-chromen-4-one	–	− 6.5	[23]
Quercetin 3,4′-dimethyl ether	5380905	− 6.5	[23]
Kirenol	15736732	− 6.2	[8]
Ferulic acid	445858	− 5.6	[35]
Succinic acid (f)	1110	− 4.5	[35]
Heptacosanol (f)	74822	− 3.5	[35]

f indicates that the compound failed to interact with the known receptor based on docking simulation

**Table 3:** Docking results of compounds from *Sigesbeckia glabrescens* Makino (SG) with p65

Name	Pubchem_ID	Affinity (kcal/mol)	References
NPDAQZUAWDTCIZ-UHFFFAOYSA-N	53462154	− 7.3	[34]
Daucosterol	5742590	− 7.2	[8]
Quercetin 3,4′-dimethyl ether	5380905	− 7.2	[23]
5-Hydroxy-2-(3-hydroxy-4-methoxyphenyl)-3,7-dimethoxy-4H-chromen-4-one	–	− 7	[23]
3,7-Di-*O*-methylquercetin	5280417	− 6.8	[23]
β-Sitosterol	222284	− 6.7	[35]
Darutoside	44715524	− 6.7	[34]
8(14)-Pimarene-2,15,16,19-tetrol;(ent-2,15S)-form,16-Ac	–	− 6.7	[36]
3-*O*-Methylquercetin	5280681	− 6.7	[23]
Darutigenol	3037565	− 6.3	[34]
Kirenol	15736732	− 6.2	[8]
Ferulic acid (f)	445858	− 5.7	[35]
Succinic acid (f)	1110	− 4.6	[35]
Heptacosanol (f)	74822	− 3.7	[35]

f indicates that the compound failed to interact with the known receptor based on docking simulation

## Discussion

The potential application of Chinese herbal medicine on the disease could be attributed to the anti-inflammatory properties [21, 22]. Therefore, we aimed to explore the anti-inflammatory effect of SG. The herb from SG was reflux-extracted with 50% ethanol and the concentrated samples were lyophilized to obtain the powdered SG extract. First, we found that the main compounds of SG, kirenol and darutoside, did not exhibit anti-inflammatory effects in LPS-induced RAW 264.7 cells. To avoid false-positive anti-inflammatory effects, we systemically evaluated the effects of the SG extract on cell number, viability, cytotoxicity, and proliferation. The comprehensive results confirmed that the cell status was normal after SG extract treatment at the following concentrations: 25, 50, 100, and 200 µg/mL. Moreover, SG exhibited potent anti-inflammatory effects at the concentration of 200 μg/mL. Therefore, the series concentrations of 25, 50, 100, or 200 µg/mL were selected for further studies on the anti-inflammatory effects of SG. We found that SG could significantly attenuate LPS-induced NO release as well as indomethacin (an inhibitor of COX-1) could. Further studies showed that SG was much more effective in inhibiting IL-6 secretion than MCP-1 secretion and TNF-α secretion.

In LPS-induced microglia, four quercetin derivatives isolated from SG were reported to exhibit anti-inflammatory activities [23, 24]. The compounds reduced LPS-induced NO production and were associated with a downregulation of COX-2 and iNOS at the protein and mRNA levels; and the compounds were also associated with the inactivation of NF-κB, p38, and the signalling of extracellular signal-regulated kinases (ERKs). The two other SG-originated diterpenoids, siegeskaurolic acid [25] and kirenol [26], were found to decrease the expression of iNOS and COX-2. Furthermore, the release of NO, PGE2, and TNF-α in LPS-induced RAW 264.7 cells was also inhibited by siegeskaurolic acid. For example, in the collagen-induced arthritis (CIA) rat model, kirenol could depress paw swelling and reduced IL-1β in the synovial fluid by upregulating nuclear annexin-1 and inhibiting the NF-κB activity [27]. In addition, SG treatment could alleviate allergic asthma by reducing the inflammatory cells in bronchoalveolar lavage (BAL) fluid and by decreasing inflammatory factors, including IL-4, IL-5, IL-13, eotaxin, and IgE, which are linked with the decreased expression of iNOS and COX-2 [28].

To further investigate the mechanism of action of SG against LPS stimulation, western blotting assays and reporter gene assays were performed to evaluate the TLR4-activated pathways. SG could not significantly regulate the MAPK pathway or COX-2. However, previous reports claimed that SG could inhibit COX-2 expression in the lung tissue of ovalbumin-challenged mice. SO (*Sigesbeckia orientalis* L.) or SP (*S. pubescens* Makino), two other species of Sigesbeckiae herba, could significantly weaken LPS- or Pam_3_CSK_4_-induced COX-2 expression in LPS- or Pam_3_CSK_4_-induced RAW 264.7 cells [29, 30]. Moreover, SO can attenuate acute inflammation by regulating inflammatory mediators via MAPK- and NF-κB-dependent pathways. Therefore, various species of Sigesbeckiae herba, containing obvious differences in chemistry, could exhibit wavering effects on different screening models [8]. Our reports also confirmed that the chemical and biological properties of SO, SP, and SG were different and distinct in anti-rheumatic mechanisms [31]. SG was shown to suppress LPS-induced iNOS expression and to downregulate LPS-induced NF-κB promoter activities, resulting in subsequent NO release. A previous report demonstrated that SG could attenuate UVB-induced photoaging in hairless mouse fibroblasts through the inactivation of the MAPK and NF-κB pathways [9]. In LPS-induced macrophages, the NF-κB pathway was modulated by SG, which was proven to inhibit the LPS-induced phosphorylation of NF-κB/p65. These results were consistent with the suppression of p65 activation by SG at the transcriptional level. KEAP1 also targets the downregulation of NF-κB activity by targeting IKK-β degradation. PIK3C3 is involved in inducing a novel autophagy pathway in macrophages that modulates inflammatory responses [32]. Our in silico data from AutoDock indicated that the SG complex may regulate the KEAP1 and PIK3C3 pathways (data not shown). Our experimental data confirmed that KEAP1 expression and PIK3C3 phosphorylation were also regulated by SG, which should be identified as having a role in cell immunity with further studies.

SG extract exhibited strong anti-inflammatory effects in the in vitro model. There are more than 40 kinds of identified components in SG, but the action of the bioactive compounds of SG is still unclear. Kirenol is a characteristic chemical constituent of SG that decreases the collagen-induced inflammation of arthritis joints through the regulation of the balance of T cells and cytokine levels via the NF-κB, Foxp3, and nuclear annexin-1 pathways [33]. Darutoside is also an important constituent of SG, but there is no evidence to support that darutoside has anti-inflammatory activity. In our screening model, kirenol or darutoside treatment alone could not reduce the LPS-induced NO release. We considered that the action of SG on inflammation may be a general effect initiated by these overall compounds of the extract rather than a single constituent.

Until now, most of the pure compounds contained in SG were not commercially available. To economically and systematically determine the underlying mechanisms of the anti-inflammatory actions of SG, molecular docking was utilized to predict the potential targets of the SG compounds. Based on the obtained results, over 10 kinds of compounds in SG were predicted to likely target TLR4, p65, and p105. However, the characteristic chemical constituent of SG, kirenol, is not the best ligand targeting protein for TLR4, p65, and p105. These results obtained from the in vitro and in silico investigations provide more evidence supporting that SG could inhibit LPS-induced inflammation by targeting TLR4 and regulating the NF-κB signalling pathway.

## Conclusions

In summary, our data indicate that SG exerts anti-inflammatory effects on RAW 264.7 cells in vitro. The underlying mechanisms of the SG anti-inflammatory properties might be involved in NF-κB modification evoked by targeting TLR4 using in silico analysis and experimental validation. SG is a potent herbal medicine for the development of new therapeutics for inflammatory diseases.

## Data Availability

Not applicable.

## Abbreviations

SG: *Sigesbeckia glabrescens* Makino
MTT: 3-[4,5-dimethyl-2-thiazolyl]-2,5-diphenyltetrazolium bromide
LDH: lactate dehydrogenase
NO: nitric oxide
NAG: *N*-acetyl-d-glucosamine
LPS: lipopolysaccharides
COX: cyclooxygenase-2
iNOS: inducible nitric oxide synthase
TNF-α: tumour necrosis factor-α
PGE2: prostaglandin E 2
MAPK: mitogen-activated protein kinase
NF-κB: nuclear factor kappa-light-chain-enhancer of activated B cells
TLRs: toll-like receptors
IκB: inhibitor κB
DMEM: Dulbecco’s modified Eagle’s medium
FBS: foetal bovine serum
PBS: phosphate-buffered saline
PS: penicillin–streptomycin
RIPA: radioimmunoprecipitation assay
ATCC: American Type Culture Collection
ERKs: extracellular signal-regulated kinases
SO: *Sigesbeckia orientalis* L.
SP: *S. pubescens* Makino
